# Diagnosis and treatment of hypoparathyroidism: a position statement from the Brazilian Society of Endocrinology and Metabolism

**DOI:** 10.20945/2359-3997000000015

**Published:** 2018-01-01

**Authors:** Sergio Setsuo Maeda, Carolina Aguiar Moreira, Victória Zeghbi Cochenski Borba, Francisco Bandeira, Maria Lucia Fleiuss de Farias, João Lindolfo Cunha Borges, Francisco José Albuquerque de Paula, Felipe Augusto Brasileiro Vanderlei, Fábio Luiz de Menezes Montenegro, Rodrigo Oliveira Santos, Bruno Ferraz-de-Souza, Marise Lazaretti-Castro

**Affiliations:** 1 Universidade Federal de São Paulo Universidade Federal de São Paulo Disciplina de Endocrinologia Departamento de Medicina São Paulo SP Brasil Departamento de Medicina, Disciplina de Endocrinologia, Universidade Federal de São Paulo (Unifesp), São Paulo, SP, Brasil; 2 Universidade Federal do Paraná Universidade Federal do Paraná Hospital de Clínicas Curitiba PR Brasil Serviço de Endocrinologia e Metabologia, Hospital de Clínicas da Universidade Federal do Paraná (SEMPR, UFPR), Curitiba, PR, Brasil; 3 Universidade de Pernambuco Universidade de Pernambuco Faculdade de Medicina Disciplina de Endocrinologia, Hospital Agamenon Magalhães Recife PE Brasil Disciplina de Endocrinologia, Hospital Agamenon Magalhães, Faculdade de Medicina, Universidade de Pernambuco (UPE), Recife, PE, Brasil; 4 Universidade Federal do Rio de Janeiro Universidade Federal do Rio de Janeiro Disciplina de Endocrinologia Departamento de Clínica Médica Rio de Janeiro RJ Brasil Departamento de Clínica Médica, Disciplina de Endocrinologia, Universidade Federal do Rio de Janeiro (UFRJ), Rio de Janeiro, RJ, Brasil; 5 Universidade Católica de Brasília Universidade Católica de Brasília Disciplina de Endocrinologia Brasília DF Brasil Disciplina de Endocrinologia, Universidade Católica de Brasília (UCB), Brasília, DF, Brasil; 6 Universidade de São Paulo Universidade de São Paulo Faculdade de Medicina de Ribeirão Preto Departamento de Medicina Interna Ribeirão Preto SP Brasil Departamento de Medicina Interna, Faculdade de Medicina de Ribeirão Preto, Universidade de São Paulo, USP, Ribeirão Preto, SP, Brasil; 7 Universidade de São Paulo Universidade de São Paulo Faculdade de Medicina Departamento de Cirurgia, Disciplina de Cirurgia de Cabeça e Pescoço São Paulo SP Brasil Departamento de Cirurgia, Disciplina de Cirurgia de Cabeça e Pescoço, Faculdade de Medicina da Universidade de São Paulo (FMUSP), São Paulo, SP, Brasil; 8 Universidade Federal de São Paulo Universidade Federal de São Paulo Departamento de Otorrinolaringologia e Cirurgia de Cabeça e Pescoço São Paulo SP Brasil Departamento de Otorrinolaringologia e Cirurgia de Cabeça e Pescoço, Universidade Federal de São Paulo (Unifesp), São Paulo, SP, Brasil; 9 Universidade de São Paulo Universidade de São Paulo Hospital das Clínicas da Faculdade Divisão de Endocrinologia e Laboratório de Endocrinologia Celular e Molecular São Paulo SP Brasil Divisão de Endocrinologia e Laboratório de Endocrinologia Celular e Molecular (LIM-25), Hospital das Clínicas da Faculdade de Medicina da Universidade de São Paulo (HCFMUSP), São Paulo, SP, Brasil

**Keywords:** Hypoparathyroidism, hypocalcemia, calcitriol, PTH, guideline, diagnosis, treatment

## Abstract

**Objective:**

To present an update on the diagnosis and treatment of hypoparathyroidism based on the most recent scientific evidence.

**Materials and methods:**

The Department of Bone and Mineral Metabolism of the *Sociedade Brasileira de Endocrinologia e Metabologia* (SBEM; Brazilian Society of Endocrinology and Metabolism) was invited to prepare a document following the rules set by the Guidelines Program of the *Associação Médica Brasileira* (AMB; Brazilian Medical Association). Relevant papers were retrieved from the databases MEDLINE/PubMed, LILACS, and SciELO, and the evidence derived from each article was classified into recommendation levels according to scientific strength and study type.

**Conclusion:**

An update on the recent scientific literature addressing hypoparathyroidism is presented to serve as a basis for the diagnosis and treatment of this condition in Brazil.

## INTRODUCTION

Serum calcium concentration is maintained within a narrow physiological range by complex controlling mechanisms involving the parathyroid hormone (PTH), active vitamin D (1,25(OH)_2_D), and calcium sensor receptors (CaSRs) acting in renal, intestinal, parathyroid, and bone tissues to maintain mineral homeostasis. When these homeostatic mechanisms fail or are not fully compensated, hypocalcemia occurs (1).

Inappropriately low (insufficient) circulating PTH levels, which in adults occurs mainly after thyroid surgery, is the most common cause of hypocalcemia. Current standard treatment of low PTH levels comprising vitamin D analogs and calcium supplementation is challenging as it does not involve replacing the missing hormone (2).

Over the past ten years, we have gained a greater understanding of hypoparathyroidism regarding its epidemiology, genetics, associated skeletal disease, and therapies. A major therapeutic challenge in hypocalcemia is effectively balancing calcium levels while avoiding hypercalciuria and other complications (3).

This document is a result of efforts by the Department of Bone Metabolism of the *Sociedade Brasileira de Endocrinologia e Metabologia* (SBEM; Brazilian Society of Endocrinology and Metabolism) for the development of recommendations based on the current evidence available in the scientific literature regarding the diagnosis and treatment of hypoparathyroidism. The objective of this document is to answer routine questions and serve as a guideline for endocrinologists and clinicians in Brazil.

## MATERIALS AND METHODS

We elaborated this guideline motivated by SBEM's Practical Guidelines Program. The model applied to this document followed the Guidelines Program of the *Associação Médica Brasileira* (AMB; Brazilian Medical Association) and *Conselho Federal de Medicina* (CFM; Federal Medical Council). After selecting collaborators with a significant role and relevant publications in the area of hypoparathyroidism, we elaborated clinical questions for discussion. We searched the databases MEDLINE/PubMed and SciELO/LILACS for relevant publications, and categorized each publication according to the level of evidence, as recommended by the Oxford Centre for Evidence-Based Medicine. These recommendations evaluate the study design and consider the best available evidence for each question to attribute a recommendation level or evidence strength to each article (4,5). In this document, we report the levels of recommendation and evidence as:

A: experimental or observational studies with consistent results.
B: experimental or observational studies with less consistent results.
C: case reports (uncontrolled studies).
D: opinion is lacking critical evaluation or is based on guidelines, physiological studies, or animal models.

## ETIOLOGY

### 1. What are the causes and differential diagnoses of hypocalcemia?

Decreases in serum ionized calcium are recognized by CaSRs in the parathyroid glands, eliciting PTH release from preexisting pools and stimulating PTH production and secretion. Serum calcium levels are then restored by PTH-mediated decreases in urinary calcium excretion, and increases in bone resorption and intestinal calcium absorption, the later in association with increased 1,25(OH)_2_D (calcitriol) synthesis in the renal tubules. Thus, the causes of hypocalcemia may be divided into those associated with PTH deficiency or resistance (addressed separately), and those not directly associated with hypoparathyroidism, as listed in Table 1 (6-9).

**Table 1:** Causes of hypocalcemia other than hypoparathyroidism and pseudohypoparathyroidism

	Total calcium	Ionic calcium	PTH	Symptoms of hypocalcemia	Bone manifestations	Associated diseases
Hypoalbuminemia	Low	Normal	Normal	Absent	None	Liver cirrhosis, nephrotic syndrome, burn, malnutrition, sepsis
Hypomagnesemia	Low	Low	Variable	Variable	None	Diuretics, chronic diarrhea, small bowel bypass or resection
Calcium deficiency	Low	Low	High	Variable	Rickets/osteomalacia	Severe malnutrition, Roux-en-Y gastric bypass (RYGB)
Vitamin D deficiency	Low	Low	High	Variable	Rickets/osteomalacia increased resorption (decreased density)	Decreased sun exposure, obesity, dark skin, RYGB, aging, severe malnutrition, celiac disease, pancreatic diseases, steatorrhea, antiepileptic drugs
Hyperphosphatemia (acute)	Low	Low	Variable	Variable	Dependent on the etiology	Phosphate-containing enemas, renal failure, intense tissue breakdown (rhabdomyolysis or tumor lysis)
Chronic kidney disease (CKD) and GFR < 30 mL/min/1.73 m^2^	Low	Variable	High	Variable	Hyperparathyroidism (eventual low turnover)	Severe hypertension, diabetes mellitus, and others
Hungry bone syndrome	Low	Low	Variable	Moderate/severe	Hyperparathyroidism	Surgical cure of hyperparathyroidism (primary or secondary to CKD)
Drugs: Antiresorptives (bisphosphonates, denosumab), calcimimetics (cinacalcet), calcium chelators (EDTA, citrate, foscarnet), antiepileptics (phenytoin, phenobarbital, carbamazepine), proton pump inhibitors, loop diuretics, chemotherapeutic drugs	Low	Low	Variable	Variable	Dependent on the etiology	Osteoporosis, primary or secondary hyperparathyroidism, blood transfusion, epilepsy, peptic ulcer, neoplasias, etc.
Acute pancreatitis	Low	Low	High	Variable	None	Alcohol abuse, gallbladder stones

Although hypoalbuminemia is the most common cause of low serum total calcium levels, it has no effect on the ionized calcium fraction and, therefore, no clinical significance. Thus, measurement of serum albumin is always recommended during the investigation of hypocalcemia, along with correction of the total calcium values, which is achieved by adding 0.8 mg/dL to the total calcium level for each 1.0 g/dL decrease in albumin below 4.0 g/dL or by the formula: Calcium corrected = Calcium measured + [(4.0 - albumin) x 0.8] (6-9).

Severe hypomagnesemia decreases PTH secretion and increases resistance to PTH effects in bone and kidney. The occurrence of hypomagnesemia should be considered in all patients with hypocalcemia and low or inappropriately normal PTH levels (6-9).

Severe calcium and/or vitamin D deficiency could be associated with hypocalcemia and lead to secondary hyperparathyroidism. Measurement of plasma 25-hydroxyvitamin D [25(OH)D], the main vitamin D metabolite stored in the body, is recommended. The active metabolite of this vitamin [1,25(OH)_2_D] has a short plasma half-life and does not reflect the vitamin D status (6-14).

In acute pancreatitis, the action of pancreatic lipase generates free fatty acids that avidly chelate the insoluble calcium salts present in the pancreas, resulting in calcium deposition in the retroperitoneum (7).

In acute hyperphosphatemia, phosphate binds avidly to calcium leading to calcium deposition, mostly in bone but also in extraskeletal tissues. Hypocalcemia is commonly found in patients with chronic kidney disease in association with low 1,25(OH)_2_D levels and secondary hyperparathyroidism (15).

The hungry bone syndrome may occur after surgical cure of severe hyperparathyroidism, leading to hypocalcemia and hypophosphatemia due to a rapid increase in skeletal mineralization. Hypocalcemia is directly associated with the severity of bone disease and concomitant vitamin D deficiency (16).

Intravenous bisphosphonates (17) and subcutaneous denosumab (18), potent antiresorptive agents used to treat osteoporosis, may lead to clinical hypocalcemia, mainly in vitamin D deficient patients. This side effect of antiresorptive agents is most commonly seen in high bone turnover states such as Paget's disease of bone.

Several other drugs may aggravate hypocalcemia by acting through diverse mechanisms, and their concomitant use should be evaluated in patients with low calcium levels. Some anticonvulsants accelerate the breakdown of vitamin D, limiting bone mineralization. Proton pump inhibitors and H_2_ blockers reduce the production of gastric acid, interfering with calcium absorption. Loop diuretics and glucocorticoids induce hypercalciuria; glucocorticoids also have detrimental effects in the intestinal action of vitamin D. Antiviral drugs have also been associated with a negative impact on calcium, vitamin D, and bone metabolism. A review of the subject by Liamis and cols. is recommended (19).

**Highlights SBEM:** In normal conditions, about 50% of the total calcium circulates bound to albumin. Low albumin concentrations may inaccurately indicate hypocalcemia, but the ionized calcium fraction is normal. Thus, total calcium concentration should always be corrected by albumin level during the investigation of hypocalcemia **(A)**.

### 2. What are the causes of hypoparathyroidism?

From a functional point of view, hypoparathyroidism arises from an inability of the parathyroid glands in secreting PTH and/or impaired PTH action, directly impacting the homeostasis of calcium and phosphorus (Table 2). Hypoparathyroidism resulting from peripheral resistance to PTH action is known as pseudohypoparathyroidism. Thus, the term “hypoparathyroidism” is commonly reserved to describe a situation in which the parathyroid glands are unable of properly producing PTH **(A)** (1-3,20).

**Table 2:** Causes of hypoparathyroidism

Hypoparathyroidism due to PTH deficiency	Parathyroid destruction	Following cervical surgery
		Cervical radiotherapy or radioiodine therapy
		Autoimmune: isolated or associated with polyglandular disease [*AIRE*]
		Parathyroid infiltration by neoplastic, granulomatous, or storage diseases (Wilson's disease and hemochromatosis)
		Riedel's thyroiditis
	Genetic diseases affecting parathyroid development and/or PTH production	Isolated hypoparathyroidism [*PTH*, *GCM2*, *SOX3*]
		Genetic syndromes associated with hypoparathyroidism (e.g. DiGeorge, CHARGE, Kenny-Caffey, Sanjad-Sakati, HDR, etc.) [*TBX1*, *NEBL*, *GATA3*, *TBCE*, *FAM111A*, *CHD7*, *SEMA3E*]
		Mitochondrial diseases
	Regulation change in PTH secretion	Autosomal dominant hypocalcemia type 1 [*CASR*] and type 2 [*GNAS11*]
		Activating anti-CASR antibodies
		Chronic hypomagnesemia and hypermagnesemia
Functional hypoparathyroidism due to peripheral resistance to PTH action	Pseudohypoparathyroidism	Genetic defects in PTH post-receptor signaling [*GNAS*, *STX16*, *NESP55*, *PRKAR1A*, *PDE4D*]

Genes associated with hypoparathyroidism are shown in square brackets. Compiled from (23,31,32).

#### 2.1. Causes of hypoparathyroidism

The main cause of hypoparathyroidism is the surgical destruction of the parathyroids **(A)**. Autoimmunity is considered the second most frequent etiology of hypoparathyroidism (1,2,21-23). Rare causes of hypoparathyroidism, such as parathyroid destruction by neoplastic infiltration or heavy metals, irradiation, radioiodine therapy, Riedel's thyroiditis and genetic diseases affecting the development of the parathyroids and/or the production of PTH are listed in Table 2. Hypoparathyroidism may also result from deregulation of PTH secretion secondary to disorders of magnesium homeostasis or abnormal activation of CaSRs due to a genetic or autoimmune cause (23).

##### 2.1.1. Postsurgical hypoparathyroidism

Surgical manipulation of the anterior cervical region is the most frequent cause of hypoparathyroidism, corresponding to 75% of the cases of the acquired form of the disease **(A)** (2,22-24). The destruction of the parathyroids may occur due to an aggressive surgical treatment of cervical cancer or may be accidental as a result of truncal ligation of the inferior thyroid arteries or inadvertent removal of the parathyroids (25-27). Relevant aspects of surgical-related hypoparathyroidism will be discussed separately in Section 4.

##### 2.1.2. Autoimmune hypoparathyroidism

Autoimmune aggression to the parathyroids is considered the second most common cause of hypoparathyroidism in adults **(A)** and may occur as an isolated endocrinopathy or as part of the autoimmune polyglandular syndrome type 1 (APECED) (2,22). Isolated autoimmune hypoparathyroidism has been related to antiparathyroid and anti-CaSR antibodies, but the pathogenic role of these antibodies is still poorly characterized. The prevalence of antiparathyroid and anti-CaSR antibody positivity in individuals with suspected autoimmune hypoparathyroidism is variable (between 25 to 40%), and the measurement of these antibodies is generally limited to research studies (2,28). In clinical practice, the presence of other autoimmune manifestations helps the identification of autoimmune hypoparathyroidism in individuals who develop nonsurgical hypoparathyroidism **(D)**. The autoimmune polyglandular syndrome type 1 is a rare autosomal recessive disease caused by mutations in the *AIRE* gene, characterized mainly by mucocutaneous candidiasis, hypoparathyroidism, and adrenal insufficiency; several other autoimmune manifestations may also occur **(C)** (29).

#### 2.2. Hypoparathyroidism due to deregulation of PTH secretion

Changes in magnesemia may lead to functional hypoparathyroidism (2,21). Magnesium participates in both processes of PTH secretion and action through the adenylcyclase system. Thus, conditions of chronic magnesium depletion (chronic diarrhea, alcoholism, poorly controlled diabetes mellitus, and chronic use of proton pump inhibitors and diuretics) cause hypocalcemia with inappropriately normal or overtly low PTH levels. On the other hand, acute hypermagnesemia (due to the excessive parenteral administration or renal insufficiency) also leads to hypocalcemia since magnesium may activate CaSRs and suppress PTH secretion (22). Rare genetic defects of factors involved in the homeostasis of magnesium (*TRMP6*, *CLND16*, *CLDN19*) may also cause hypoparathyroidism (22). Therefore, serum magnesium levels must be determined upon evaluation of hypocalcemia, to exclude functional hypoparathyroidism **(A)**. PTH secretion may also be deregulated by anti-CaSR antibodies activating the CaSR receptor by mimicking calcium and inhibiting the secretion of this hormone; this is a rare cause of hypoparathyroidism (28).

##### 2.2.2 Causes of pseudohypoparathyroidism

Peripheral resistance to PTH action resulting in functional hypoparathyroidism is known as pseudohypoparathyroidism. This condition is caused by genetic defects in postreceptor PTH signaling and is characterized in laboratory tests by hypocalcemia and hyperphosphatemia in the presence of elevated PTH levels in patients with normal renal function **(A)**. In pseudohypoparathyroidism, the production of PTH by the parathyroids is normal, and the biochemical disorder resulting from hormonal resistance (hypocalcemia and hyperphosphatemia) stimulates increased PTH production. The main genetic defects causing pseudohypoparathyroidism are inactivating mutations of the alpha subunit of the stimulatory G protein, coded by the *GNAS* gene, which in physiological conditions acts by coupling to the PTH receptor and propagating the stimulation arising from the binding of the hormone to the receptor **(A)** (30). Thus, in pseudohypoparathyroidism, defects in the *GNAS* gene interfere with PTH signaling in peripheral target tissues, particularly in the kidneys. Other molecular defects (for example, modification in *GNAS* methylation) or defects in other mediators of PTH signaling in target tissues (for example, PRKAR1A) may also cause pseudohypoparathyroidism **(A)** (23). The diagnosis and management of pseudohypoparathyroidism are beyond the scope of this article.

**Highlights SBEM:** In patients without a history of conditions leading to parathyroid destruction, other causes of hypocalcemia must be considered. Clinical and laboratory evaluation of these patients is fundamental since, in most cases, it is not difficult to identify the cause of the hypocalcemia **(A)**, although differentiating autoimmune from idiopathic hypoparathyroidism may be clinically challenging.

## EPIDEMIOLOGY

### 3. What is the prevalence of hypoparathyroidism?

Hypoparathyroidism is a rare disorder with an estimated prevalence of 0.25 per 1,000 individuals **(B)** (33,34). Most patients with hypoparathyroidism had their parathyroids incidentally removed or injured during thyroid surgery (35). Transient postsurgical hypoparathyroidism is common, due to functional parathyroid impairment after acute manipulation, with subsequent spontaneous recovery. However, it may, more rarely, be definitive **(C)** (22). Despite literature reports of recoveries occurring more than six months after surgery, hypoparathyroidism is considered definitive when lasting more than six months from the surgical event **(D)** (2,3,20,36).

The actual prevalence of postsurgical hypoparathyroidism is probably underestimated, due to lack of clear definitions, inappropriate follow-up, conflicts of interest in reports of individual series, and different strategies in calcium and vitamin D supplementation, among other causes. Thus, the prevalence of transient hypoparathyroidism varies widely from 3% to 52%, while that of definitive hypoparathyroidism ranges from 0.4% to 13% (24,37-44). Since postsurgical hypoparathyroidism is due to direct parathyroid injury, treatment with total thyroidectomy is associated with increased rates of hypoparathyroidism when compared with partial thyroid surgeries (subtotal thyroidectomy and hemithyroidectomy) (Table 3). On the other hand, these rates are even higher with central compartment (level VI) lymph node dissections and in thyroid and parathyroid reoperations, especially in parathyroid hyperplasia **(B)** (45,46). Although the Brazilian literature in this regard is scarce, the results of Brazilian studies are aligned with those described above (25,47-52) (Table 3).

**Table 3:** Epidemiology of post-surgical hypoparathyroidism

Reference	Author, year	Country	Prevalence of hypoparathyroidism	Comments
27	Underbjerg and cols., 2013	Denmark	22/100,000 (general population)	Data from 1988 to 2012
33	Powers and cols., 2013	USA	5.0%	120,000 surgeries in one year
35	Rosato and cols., 2004	Italy	1.7%	15,000 thyroidectomies with a 5-year follow-up
37	Youngwirth and cols., 2010	USA	0.74%	12% of patients had transient hypoparathyroidism, only 2 required calcitriol in the long term
38	Snyder and cols., 2013	USA	1.0% (for Graves’ disease) 1.8% (for other types of hyperthyroidism) 0% (for other benign diseases)	780 total thyroidectomies for different reasons
39	Pelizzo and cols., 2014	Italy	3.3%	233 patients after a second thyroid surgery
40	Puzziello and cols., 2014	Italy	0.9%	2,631 thyroidectomies 28.8% had transient hypocalcemia
41	Ritter and cols., 2015	USA	1.9%	1,054 thyroidectomies
42	Wei and cols., 2014	China	0.9% (3/321) – autotransplantation 3.8% (6/156) – parathyroid glands preserved in place	477 thyroidectomies with (n = 321) and without (n = 156) parathyroid autotransplantation
43	Ito and cols., 2014	Japan	8%	154 completion thyroidectomies after a previous thyroidectomy
44	Lorente-Poch and cols., 2015	Spain	4.6%	657 thyroidectomies, 1-year follow-up
45	Nawrot and cols., 2014	Poland	8.5%	401 thyroidectomies
46	Järhult and cols., 2012	Sweden	6% of the total thyroidectomies	265 thyroidectomies in patients with Graves’ disease
47	Dedivitis and cols., 2010	Brazil	6.6%	91 thyroidectomies, 6 patients had biochemical hypoparathyroidism after 1 month
48	Accetta and cols., 2011	Brazil	0/66 – no case	66 thyroidectomies
49	Vanderlei and cols., 2012	Brazil	2.5%	40 thyroidectomies
50	Molinari and cols., 2015	Brazil	0.3%	3,411 thyroidectomies
51	Ywata de Carvalho and cols., 2015	Brazil	11.8% of patients with central neck dissection 2.3% of patients without central neck dissection	Two different groups: total thyroidectomy with (n = 106) and without central neck dissection (n = 478)
52	Montenegro and cols., 2012	Brazil	28%	83 patients with hyperparathyroidism due to MEN-1/all with parathyroid autotransplantation

**Highlights SBEM:** There are no consistent data on the prevalence of permanent hypoparathyroidism after surgery. The risk of postsurgical hypoparathyroidism should be considered upon recommendation of thyroid/parathyroid surgeries, along with the extent of the procedures **(C)**.

## SURGICAL ASPECTS

### 4. Is it possible to prevent postsurgical hypoparathyroidism?

Injury to the parathyroid glands during thyroid surgery may be due to direct tissue trauma (mechanical or thermal), injury to the vascular pedicle, inadvertent removal of the parathyroids during surgery, or even intentional removal of the glands for oncologic reasons (for example, presence of metastases in the central compartment, which upon resection could also remove the parathyroids from their location) (53,54).

Maintaining intact parathyroids in place is the most important factor in preventing hypoparathyroidism (44,55). This requires from the surgeons a broad knowledge of the anatomy and embryology of the parathyroids, meticulous surgical technique, and command of parathyroid autotransplantation techniques in case the glands are removed (43,56,57).

There is no standard and widely used technique in terms of handling of the parathyroids during thyroidectomy, but some aspects may be highlighted: ligation of the inferior thyroid artery branches should be performed as close as possible to the thyroid capsule to avoid vascular lesions; direct cauterization of the parathyroids or within millimeters from them must be avoided; careful dissection of the thyroid capsule should be performed, gently moving away the parathyroids without causing tissue or vascular trauma; when central compartment lymph node dissection is required (where the inferior parathyroids are at greater risk), start by identifying and maintaining the superior parathyroids intact and in place; inspect the thyroid after removal in search of parathyroids that may have been inadvertently resected. In this case, autotransplantation of small fragments of the removed parathyroid (approximately 2 mm^3^) is recommended, followed by their implantation usually in the homolateral sternocleidomastoid muscle (43,56,57), although the actual effectiveness of this procedure has been questioned by several authors **(B)** (44,58).

There have been recent attempts at the development of auxiliary methods to identify the parathyroid glands during thyroidectomy, such as the injection of nanoparticles of carbon into the thyroid (59,60) and detection of the natural autofluorescence of the parathyroid with near-infrared imaging (61-63) or in association with methylene blue (64) or indocyanine green (65). In spite of these techniques improving the identification of the parathyroids, they have been unable to reduce the rates of hypoparathyroidism after total thyroidectomy **(B)** (66).

**Highlights SBEM:** The maintenance of intact parathyroids in place is the most important factor in preventing hypoparathyroidism. Extensive knowledge of anatomy and embryology, meticulous surgical technique, and command of parathyroid autotransplantation techniques may minimize the risk of hypoparathyroidism. To date, no technology for intraoperative identification of the parathyroids has proven effective in reducing the rates of permanent hypoparathyroidism **(B)**.

### 5. Is it possible to predict the risk of hypocalcemia after thyroidectomy?

Efforts have been directed toward the identification of patients at higher risk of developing hypocalcemia after thyroidectomy. These efforts were initially focused on protocols recommending serial blood sample collection for measurement of calcium levels (67-69) and, more recently, also include measurement of PTH levels (49,70-85). The literature describes several protocols for measurement of serum PTH levels (49,73,80,82), with variations in the time of blood collection, criteria for the diagnosis of hypocalcemia, number of collected blood samples, and different PTH cutoff levels. The decay of PTH levels in samples collected before and 1 hour after surgery has been associated with the occurrence of hypocalcemia **(B)** (49,83). A single postoperative measurement has also shown to correlate with the occurrence of hypocalcemia. Samples can be collected from the end of the surgery (49,70,71,78,79,81) till the first postoperative day **(B)** (72,76,84). None of these studies has guaranteed 100% accuracy with such measurements. Severe hypocalcemia is unlikely with a normal PTH value measured after surgery (75). Although the time period, PTH value, and ideal protocol have not been defined yet, perioperative PTH measurements appear to be useful in the postoperative management of patients undergoing thyroidectomy who later develop hypoparathyroidism.

**Highlights SBEM:** The use of perioperative PTH values may be useful in predicting the risk of hypocalcemia in patients undergoing thyroidectomy. However, the protocol must be established according to the experience of each institution **(B)**.

## CLINICAL MANIFESTATIONS

### 6. What are the acute and chronic clinical manifestations of hypocalcemia?

Hypocalcemia may be associated with several signs and symptoms, considering that calcium has distinct roles in various organs and tissues, including the central nervous system, heart, skeletal muscle, and kidneys (2,3,22). The severity of the symptoms depends on the duration and intensity of the hypocalcemia and on how fast the manifestations emerge **(D)**. Classically, low serum calcium manifests acutely with paresthesias in the face or distal extremities, weakness, and muscle pain accompanied or not by increased levels of the enzyme creatine phosphokinase (CPK) **(C)** (86,87). Hypocalcemia is associated with increased neuromuscular excitability; therefore, symptoms such as carpopedal spasms, cramps, and muscle contraction are characteristic of this metabolic disorder.

On physical examination, the characteristic signs of hypocalcemia reflecting a status of neuromuscular excitability are Trousseau's and Chvostek's signs. The first is an involuntary contraction of the forearm muscles with flexion of the wrist and metacarpophalangeal joint, extension of the interphalangeal joints, and adduction of the thumb (“obstetrician's hand” or carpopedal spasm) when the cuff of the sphygmomanometer is inflated 10 to 20 mmHg above the systolic pressure for about 3 minutes causing an occlusive pressure (88). Although this sign is very specific of hypocalcemia, it may also be present in up to 4% of the normal individuals **(B)**. The Chvostek's sign is characterized by an ipsilateral contraction of the muscles around the lips or other facial muscles triggered by the tapping of the facial nerve over its trajectory anterior to the ear (89). This signal may be also positive in up to 10% of the normal individuals **(D)**.

In severe cases, excessive muscle contraction may lead to spontaneous tetany or trigger generalized seizures, which may be the initial presentation of hypoparathyroidism **(C)** (90). In addition, acute and severe hypocalcemia can potentially lead to sudden dyspnea followed by laryngeal stridor, characterizing laryngospasm (91), or result in papilledema associated or not with increased intracranial pressure. All these situations are reversible with normalization of serum calcium levels **(C)** (92,93).

Since calcium plays a fundamental role in myocardial excitation and contraction, severe hypocalcemia triggers electrocardiographic changes such as prolongation of the QT interval, which may progress to ventricular fibrillation and cardiac arrest **(C)** (94). There have been reports of dilated cardiomyopathy with systolic dysfunction reversing after normalization of calcium levels **(C)** (95,96).

Some dermatological manifestations secondary to acute hypocalcemia have been reported, including piodermitis and acute generalized pustular psoriasis (psoriasis of von Zumbusch) **(C)** (97-99). The reason for the association between hypocalcemia and psoriasis, specifically, is the role of the intracellular calcium in regulating the proliferation and differentiation of keratinocytes (97-99).

In contrast to the manifestations in acute hypocalcemia, neuromuscular symptoms may be milder in chronic hypocalcemia **(D)** (2,3,22). Some chronic manifestations such as cataract and cerebral calcification will be discussed in the session dedicated to chronic complications (100-102).

Hypocalcemia of long duration has been associated with psychiatric manifestations, such as mood changes, anxiety, depression, and, more rarely, hallucinations and psychotic episodes **(C)** (103).

Other symptoms including tinnitus and dizziness have been reported in patients with hypocalcemia and cerebral calcification **(C)** (104). Nonspecific signs may be present, such as dry and rough skin, weak and brittle nails, and dry hair (105). Dental abnormalities, especially during childhood, such as delayed teeth eruption, dental hypoplasia, and enamel and tooth root defects, have also been reported **(C)** (106).

**Highlights SBEM:** The most frequent symptoms of acute hypocalcemia are paresthesias, cramps, pain, and muscle weakness. In cases of severe hypocalcemia, seizures, tetany, papilledema, and laryngospasm may be included in the clinical manifestations. On physical examination, the presence of Trousseau's and Chvostek's signs suggest hypocalcemia. Chronic hypocalcemia may be asymptomatic **(C)**.

## DIAGNOSIS

### 7. How is hypoparathyroidism diagnosed?

Although hypoparathyroidism is suspected on clinical grounds, its diagnosis is based on laboratory tests demonstrating inappropriately low PTH levels in the presence of hypocalcemia **(A)** (1-3,9,21). Of note, the definite calcium and PTH values for diagnosis of hypoparathyroidism have not been defined yet.

The diagnosis of hypoparathyroidism should take into account the presence of suggestive clinical manifestations, history of surgery or cervical irradiation, and factors that might suggest the etiology of the disease, such as concomitant autoimmune conditions or syndromic manifestations (2,20). The physical examination must include a careful inspection of the cervical region in search of signs of prior surgery and assessment of Chvostek's and Trousseau's signs (1-3).

The laboratory evaluation must include serum measurement of total calcium corrected for albumin, PTH, phosphorus, magnesium, creatinine, and 25(OH)D, in addition to 24-hour urinary calcium **(A)** (1-3,9,21).

Some laboratory features of hypoparathyroidism are noteworthy. Although ionized calcium is the physiologically active calcium fraction, its measurement requires standardized blood collection and pre-analytical care, both of which are not always followed. In clinical practice, the most reliable alternative to ionized calcium measurement is the determination of total calcium corrected for albumin, which is calculated by adding 0.8 mg/dL for each 1.0 g/dL decrease in albumin level below 4.0 g/dL (6-9). The diagnosis of hypocalcemia is established in the presence of calcium levels below the normal range, but in hypoparathyroidism, the levels are usually below 7.5 mg/dL **(C)**. In cases of rapid development, the symptoms may precede the hypocalcemia **(C)** (107).

The values of intact PTH are low or undetectable. In the presence of hypocalcemia, values below 20 ng/mL are diagnostic of hypoparathyroidism (20). PTH measurement requires certain care with blood collection and storage (108).

Levels of phosphorus in chronic hypoparathyroidism are usually increased, but in the presence of concomitant hungry bone syndrome, they may be within the normal range or decreased. Measurement of magnesium levels is important to rule out functional hypoparathyroidism. The determination of serum creatinine and 25(OH)D levels are useful during follow-up.

In the presence of a normal renal function, the 24-hour urinary calcium reflects the nutritional intake of calcium. During follow-up, it is important to monitor the occurrence of hypercalciuria (urinary calcium greater than 4 mg/kg/day), due to the absence of PTH during treatment with calcium and vitamin D **(A)** (109,110).

The etiologic diagnosis of nonsurgical hypoparathyroidism is challenging. So far, no autoantibody has been standardized for this purpose in clinical practice, although the determination of anti-IFNω and anti-NALP5 has proven to be useful in this regard (111). The diagnosis of autosomal dominant hypocalcemia through molecular CaSR analysis is important during management of this condition to prevent nephrolithiasis/nephrocalcinosis **(B)** (111).

**Highlights SBEM:** Hypoparathyroidism is suspected on clinical grounds, but its diagnosis is based on laboratory tests indicating inappropriately low PTH levels in the presence of hypocalcemia **(A)**. The patient's laboratory evaluation must include total serum calcium corrected for albumin, PTH, phosphorus, magnesium, creatinine and 25-hydroxyvitamin D, in addition to 24-hour calciuria **(A)**.

## TREATMENT

### 8. How should patients with hypoparathyroidism be treated? How should therapeutic failure with conventional treatment be defined?

Treatment of hypoparathyroidism is aimed at correcting hypocalcemia and hyperphosphatemia, reducing symptoms, and preventing chronic complications resulting from the disease or its treatment. To avoid long-term side effects, the goal of the treatment is to maintain the total calcium close to the lower normal range. Persistent hypomagnesemia after normalization of calcium levels must be corrected (112). Two different strategies may be used, depending on the rate of development of hypocalcemia and presence of symptoms **(A)**.

#### 8.1 Treatment of acute hypocalcemia

Acute manifestations threatening the patients’ lives such as tetanic seizures, laryngospasm or bronchospasm, seizures, bradycardia, prolongation of the QT interval, or congestive heart failure, require urgent treatment with intravenous calcium **(A)** (1-3,7,20,21,107,112,113). The most used salt among us is 10% calcium gluconate, which contains approximately 90 mg of elemental calcium per 10 mL of solution. One to two ampoules should be diluted in 50 to 100 mL of 0.9% saline solution (SS) or 5% glycosylated solution (GS) and administered slowly via intravenous infusion over 10 to 20 minutes **(B)**. Rapid infusion may trigger cardiac arrhythmias and cause a severe inflammatory reaction in the venous path (phlebitis). Overflow may lead to calcification of local soft tissues, especially when serum phosphorus concentrations are increased. To preserve normocalcemia, a continuous infusion of elemental calcium 0.5 to 2.0 mg/kg of body weight/hour diluted in 5% GS may be required until the effect of the long-term oral medications on calcium levels becomes established **(B)**. Serum calcium concentrations must be periodically monitored for titration of the infusion dose; the heart rhythm should also be monitored, especially in patients on digitalis **(A)** (1-3,7,20,21,107,112,113).

#### 8.2 Treatment of chronic hypocalcemia

Calcium concentrations must also be corrected in patients with slowly developing hypocalcemia (chronic), but intravenous calcium infusion is not required if the patient is mildly symptomatic or asymptomatic.

Maintenance of calcium concentrations in the long term has been achieved with the use of vitamin D or its active form, calcitriol, associated with oral calcium salts **(A)** (1-3,7,20,21,107,112,113).

The conversion of 25(OH)D into its active metabolite (1,25(OH)_2_D or calcitriol) is catalyzed by the enzyme 1α-hydroxylase in renal tubular cells, usually stimulated by PTH and inhibited by hyperphosphatemia (11). Therefore, the production of 1,25(OH)_2_D is reduced in hypoparathyroidism. For this reason and due to a shorter half-life, treatment with oral calcitriol is preferable **(B)**, although vitamin D (cholecalciferol or ergocalciferol) may also be used **(B)** (114). The effects of these hypercalcemic drugs arise mainly from their action on the absorption of calcium in the intestine, but when administered in excessive doses, they stimulate bone resorption and promote the release of calcium from the bone **(A)** (115). In severe PTH deficiency, calcium levels can only be normalized with calcitriol or high doses of vitamin D **(C)**. With a long biological half-life (4 to 6 hours), calcitriol may be administered every 12 hours, and its initial doses range from 0.5 to 1.0 µg/day divided into at least two daily doses. Serum calcitriol peak is achieved 4 to 6 hours after administration, and elevations in calcium levels may be observed 1 to 3 days after treatment initiation. The dose of calcitriol should be titrated according to calcium levels and varies among individuals, in some cases exceeding 2.0 µg/day **(B**) (1-3,7,20,21,107,112,113). High doses of vitamin D (ergocalciferol or cholecalciferol) may also be used to treat hypoparathyroidism and have often been used in the past when access to calcitriol was restricted. The effects of these agents in increasing calcium levels take longer (approximately ten days), and their biological half-life is 2 to 3 weeks. Since these doses are very high, situations of severe intoxication manifesting with hypercalcemia may occur in the long term and are usually very prolonged **(A)** (114). Due to that, there is a preference for the use of calcitriol, whose shorter half-life enables faster correction of calcium levels, both in cases of hypocalcemia as well as hypercalcemia secondary to intoxication **(B)**. There is no evidence that the use of vitamin D supplementation doses associated with calcitriol is effective in controlling calcium when compared to calcitriol alone.

The use of oral calcium salts is essential in the treatment of hypocalcemia and has two objectives: to offer calcium for absorption by the intestinal cells under the effect of vitamin D, and to sequester radicals containing phosphorus present in the food, indirectly reducing phosphatemia (1-3).

The most commonly used calcium salts are carbonate and citrate. Calcium carbonate has a larger amount of elemental calcium per gram of salt (40%) and a lower cost. However, the calcium requires gastric acidity to dissociate from the salt and be absorbed. In cases of achlorhydria, low acidity (use of proton pump inhibitors), or gastrectomy, calcium citrate is preferred despite a lower concentration of elemental calcium per gram of salt (21%) and a higher cost (113). Other calcium formulations may be used, such as lactogluconate and citrate malate, but their use has limited scientific evidence. The daily amount of required elemental calcium varies greatly among patients, from as little as 1 g to as much as 9 g (21), but most of patients can be well controlled with daily doses ranging from 1 to 3 g, divided in three times a day over meals **(A)** (1-3,7,20,21,107,112,113). Due to the absence of the phosphaturic PTH action, it is recommended to limit the amount of phosphates and calcium phosphate salts in the diet. For this reason, increased intake of dairy products, which are rich in calcium but have a high phosphorus content, should not be encouraged **(D)**.

The use of intestinal phosphate chelating agents may be rarely necessary, in addition to the use of calcium carbonate, which also performs this function **(B)** (1-3). Unfortunately, vitamin D derivatives do not fully replace the effects of PTH, so this treatment is not substitutive. An important PTH effect is an increase in tubular calcium reabsorption. When calcium levels are increased during vitamin D administration, the supply of calcium to the glomerular filtrate increases. Without the effect of PTH to induce tubular calcium reabsorption, hypercalciuria may occur and lead in the long term to complications such as nephrolithiasis, nephrocalcinosis, and renal insufficiency **(A)** (24,116,117). To correct hypercalciuria during hypoparathyroidism treatment, thiazide diuretics including chlorthalidone and hydrochlorothiazide may be used, although this approach has variable efficacy. The dose of hydrochlorothiazide varies between 25 to 50 mg administered in one or two doses a day and the association with amiloride may help spare potassium, avoiding hypokalemia secondary to prolonged use of such diuretics **(B)** (1-3,7,20,21,107,112,113).

Magnesium has metabolic pathways very similar to those of calcium, and its level may decrease, especially in the occurrence of hypocalcemia. Usually, magnesium levels correct in parallel to those of calcium, but in some cases, supplementation with magnesium salts is required, particularly in hypoparathyroidism resulting from activating CaSR mutation (1-3,113). Commercially available magnesium pidolate has 130 mg of elemental magnesium in each vial and the dose varies between 1 to 2 vials a day, but manipulated formulations may also be used.

Treatment of hypoparathyroidism with calcium salts and vitamin D has some challenges. The success of the treatment depends on the patient ingesting several pills many times a day **(B)** (1-3,113).

Some clinical situations may destabilize a welladjusted treatment or even hinder a proper control of calcium levels, including gastrointestinal infections, urgent hospitalizations, medications affecting the absorption of nutrients or other medications (orlistat, cholestyramine, and glucocorticoids, among others), as well as conditions associated with spontaneous malabsorption (inflammatory bowel disease, celiac disease, or malabsorption due to other causes) or iatrogenic (such as post-bariatric surgeries) and difficulties in the ingestion of medications by mouth, as seen in wide surgical resections due to laryngeal cancer (2,118-123). The metabolic control during pregnancy and lactation in patients with hypoparathyroidism will be discussed in Section 10.

The use of PTH and its derivatives has been tested with some success in the treatment of hypoparathyroidism, and the use of PTH (1-84) has been approved by American and European agencies (FDA and EMA) and will be discussed in Section 9 (124). Furthermore, perspectives point out to the use of allografts of macroencapsulated parathyroid cells, but studies in this regard are still in experimental phases (125).

Considering that a hypocalcemia emergency may be potentially lethal, patients with chronic hypoparathyroidism must carry an identification card indicating their diagnosis and treatment, in the case of emergency **(D)**.

**Highlights SBEM:** Treatment of hypoparathyroidism aims at correcting hypocalcemia and hyperphosphatemia, reducing symptoms, and preventing chronic complications **(B)**. The treatment goal is to maintain the patient asymptomatic, with total calcium levels close to the lower normal range, phosphorus levels close to the high normal range and 24-hour urinary calcium should be maintained within the normal range (below 300 mg/day) or below 4 mg/kg/day. Treatment of acute hypocalcemia is aimed at controlling situations leading to imminent life threat and requires an intravenous infusion of calcium. The treatment of chronic hypocalcemia involves the use of active vitamin D and calcium salts **(B)**.

### 9. What evidence supports the use of PTH analogs in the treatment of hypoparathyroidism?

Hypoparathyroidism persists as the last classic hormone deficiency in which the conventional treatment is not done with replacement of the missing hormone **(D)** (1-3). There has been some accumulated experience with the use of PTH analogs since 1996, when Winer and cols. followed up adults treated with PTH (1-34,126-128) and studies with the duration of 3 years compared this type of treatment with the conventional one in children and adults (5-70 years). Serum calcium levels were more consistent, and no differences in urinary calcium were observed **(B)** (129,130). Studies with PTH (1-34) administered with a continuous infusion pump, compared with subcutaneous PTH (1-34) twice daily in adults and children, showed a more physiological control of serum calcium and reduction in urinary calcium **(B)** (131,132). It is important to highlight that PTH (1-34) is only approved for the treatment of osteoporosis in adults and for 18 to 24 months, and has not been approved by regulatory agencies for the use in hypoparathyroidism.

In January 2015, the FDA approved the use of PTH (1-84) for the treatment of hypoparathyroidism. From a pharmacokinetic standpoint, this molecule has a longer half-life, which allows for a single daily application. Studies have shown that the dose of PTH (1-84) must be titrated between 25, 50, 75, and 100 µg, thus enabling a reduction in the doses of calcium and vitamin D. Serum calcium must be monitored since episodes of hypercalcemia have been described, mostly asymptomatic, especially with the dose of 100 µg. In some cases, calcium and vitamin D supplements could be discontinued **(B)** (133-135).

The REPLACE study (a double-blind, randomized, phase 3, placebo-controlled trial) has shown that 53% of the adult patients (18-85 years) treated with up to 100 µg of PTH (1-84) were able to decrease by 50% their doses of calcium and vitamin D after 24 weeks compared with 2% in the placebo group **(A)** (136). A study with longer duration was published by Rubin and cols. with 33 patients followed up for six years with dose titration. There was a reduction in urinary calcium and maintenance of serum calcium, but a decrease in radial bone mineral density (BMD) in 33% **(B)** (124).

The use of PTH analogs to treat hypoparathyroidism is promising, but several limiting aspects must be considered: these agents must be administered by injection, are very expensive, and have not been approved in Brazil yet (until November 2017). Additionally, their efficacy in preventing the emergence of chronic complications has not been proven so far, there are no studies in children, and the supplementation with calcium and vitamin D has not been entirely suspended in most patients. However, some situations have been considered as potential indicators of benefit from their use in the USA, where the drug has been approved (113,137).

**Highlights SBEM:** PTH analogues may be used to treat hypoparathyroidism in specific cases. As far, there is no evidence showing their effectiveness in preventing chronic complications in the long term **(D)**.

### 10. How should hypoparathyroidism be managed during pregnancy and lactation?

The unique physiological environment during pregnancy, determined by cardinal transient factors such as the presence of the placenta (which is equipped with an endocrine machinery) and the demand for building blocks for the development of fetal organs and tissues (including bone), challenge adaptations in mineral metabolism. First, we must call attention to prominent differences in the consequences of vitamin D and PTH deficiencies in pregnant woman and the fetus (138,139).

Physiological adaptations during pregnancy and lactation affect the management of maternal hypoparathyroidism. Calcium absorption is facilitated during pregnancy by increased calcitriol synthesis and sensitivity, among other factors. At this phase, maternal PTH secretion is inhibited, but serum calcium corrected for albumin remains normal. In contrast, the action of the PTH-related protein (PTHrP) produced by the mammary gland tends to mobilize calcium from the skeleton during lactation (138,139).

Another important consideration in the management of hypoparathyroidism during pregnancy is the effect of hemodilution on serum albumin levels and circulating total calcium. Due to that, ionized calcium is the preferred control parameter and should be measured every 2-3 weeks. However, the technique to measure ionized calcium is very sensitive and requires rigid but often neglected pre-analytical protocols, leading to inaccurate results (140). In this context, the determination of total calcium corrected for albumin is an advisable alternative.

Hypoparathyroidism is a risky condition affecting the maternal/neonatal survival (spontaneous abortion, stillbirth, and premature labor), and impairing fetal bone development. Hypocalcemia in pregnant women with poorly controlled hypoparathyroidism leads to fetal secondary hyperparathyroidism with devastating effects on fetal bone, expressed as bone demineralization and fractures. In this case, concomitant maternal hyperphosphatemia is an additional worsening factor **(C)** (138,139).

The literature about the management of hypoparathyroidism during pregnancy and lactation is scarce and mostly based on case reports without randomized controlled studies comparing optional therapies (2). Frequent evaluations are necessary to assess symptoms and serum levels of albumin-corrected total calcium. Circulating calcium levels must be maintained in the lower normal range. During lactation, serum calcium levels must be closely verified due to the production of PTHrP by the breast and increased bone resorption, while medication tapering usually becomes necessary **(C)** (141).

Activated vitamin D analogs (calcitriol or 1 α-calcidiol) are preferable, but when unavailable, pharmacological doses of D_2_ or D_3_ may be used. The occurrence of an alternate source (placental) of 1,25(OH)_2_D and PTHrP during pregnancy indicate a potential need for lowering the medication dose. However, there is usually no need to reduce the doses of calcium and calcitriol in clinical practice. Indeed, the increased demand of calcium for skeletal mineralization in the last trimester increases the requirement of calcitriol to avoid hypocalcemia. In case reports, oral elemental calcium varies from 800 to 1,500 mg at the beginning of pregnancy to 2,000 to 3,200 mg in the third gestational trimester **(C)** (142,143).

During pregnancy, the daily recommended dose of calcitriol is between 0.25-3 µg. At the beginning of lactation, the dose has to be individually reduced to avoid hypercalcemia. After weaning, the woman usually resumes the medication dose used before pregnancy.

Pediatricians must be advised about the management of maternal hypoparathyroidism during pregnancy. When maternal overtreatment results in hypercalcemia, which in turn suppresses fetal PTH secretion, neonatal hypocalcemia must be assessed. On the other hand, maternal hypocalcemia may lead to fetal secondary hyperparathyroidism and skeletal abnormalities.

The authors encourage the readers to review the insightful articles by Kovacs and cols. about mineral metabolism in this singular period of a woman's life (138,139).

**Highlights SBEM:** Treatment of hypoparathyroidism during pregnancy and lactation requires special care and more frequent control for dose adjustment, which must be individualized **(C)**.

## COMPLICATIONS

### 11. What are the chronic complications of hypoparathyroidism? How should they be monitored?

The chronic complications of hypoparathyroidism are associated with the progression of the disease and the implemented therapy and include clinical comorbidities of varying severity **(D)** (22). Since for many years hypoparathyroidism remained a neglected disease, data on the long-term complications of this condition are rare.

#### 11.1 Renal manifestations

Renal complications arise from chronic hypocalcemia and hyperphosphatemia due to lost regulation of calcium and phosphorus metabolism **(D)** (144). Treatment with large amounts of calcium and active vitamin D (calcitriol) leads to hypercalciuria, in addition to increasing the intestinal absorption of phosphorus, intensifying the hyperphosphatemia and increased calcium-phosphorus product and predisposing to nephrolithiasis and nephrocalcinosis. In a study by Mitchell and cols. in patients with hypoparathyroidism, higher serum calcium levels were associated with higher urinary calcium values, prevalence of 38%, while lithiasis and nephrocalcinosis were observed in 31% of the patients. Renal function was decreased in 52% of the patients and was associated with age, disease duration, and proportion of time with relative hypercalcemia **(A)** (116). In a study by Lopes and cols., the prevalence of renal complications in patients with hypoparathyroidism was 25%; although the levels of urinary calcium were within the normal range in most of the cohort, the levels in the group with renal complications were higher (around 3.3 mg/kg/day) (24). Renal manifestations occur independently from the etiology of the hypoparathyroidism (34,27) and are already present at birth in children affected with this disorder (117).

Although it is unclear in the literature whether prophylactic measures can prevent renal impairment, measurement of urinary calcium and serum creatinine are recommended every six months. The 24-hour urinary calcium should be maintained within the normal range for gender (below 300 mg/day) (21,109) or below 4 mg/kg/d (20,110). Periodic imaging evaluations of the kidney and urinary tract are not supported in the literature. However, based on the high prevalence of renal manifestations, both European and American consensus recommendations include periodic imaging evaluations **(D)** (2,20).

#### 11.2 Cardiovascular manifestations

Two studies with historical and controlled cohorts in patients with nonsurgical hypoparathyroidism and postsurgical hypoparathyroidism have addressed the occurrence of cardiovascular complications. Although the general mortality in these cohorts was not increased, ischemic heart disease, stroke, and arrhythmia were more frequent in patients with nonsurgical hypoparathyroidism compared with the general population **(A)** (34). These complications should be individually monitored, and no established routine has been recommended.

#### 11.3 Ocular manifestations

Epidemiological studies have shown that patients with nonsurgical hypoparathyroidism have a fourfold increased risk and earlier onset of cataract when compared with the general population **(A)** (145). Annual clinical monitoring and metabolic control are recommended, despite the lack of studies confirming an advantage of routine assessment in patients with hypoparathyroidism **(D)** (2).

#### 11.4 Neuropsychiatric manifestations

Chronic complications of hypoparathyroidism affect the central nervous system and include cerebral calcifications, decreased seizure threshold, seizures, depression, and decreased quality of life (146). Cerebral calcifications vary in prevalence from 12% to 74% and are associated with the duration of the hypocalcemia **(A)** (103). Regardless of the etiology of the hypoparathyroidism, the occurrence of seizures is frequent and associated with a greater risk of hospitalization **(A)** (34,145). Therefore, determination of serum calcium is recommended during the etiological investigation of all patients with epilepsy **(D)**.

Decreased quality of life is a common and important chronic complication of hypoparathyroidism and is associated with a multifactorial etiology. Although undervalued, studies report that decreased quality of life affects 32% to 65% of the patients with hypoparathyroidism **(A)** (34,103). Physical complaints (muscle spasms, decreased muscle strength, fatigue, myalgia, and paresthesia), cognitive symptoms (“brain fog” and difficult concentration), and depression and/or anxiety are associated with decreased quality of life **(D)** (22). Depression and affective disorders are twice as frequent in postsurgical hypoparathyroidism and are associated with a feeling of poor health **(A)** (147). In nonsurgical hypoparathyroidism, there is a higher risk of hospitalization due to psychiatric diseases and a tendency to depression **(A)** (145). Traditional treatment of hypoparathyroidism is unable to prevent these manifestations **(B)** (18), and results obtained with PTH replacement show discrepancies between studies **(B)** (148,149).

#### 11.5 Musculoskeletal manifestations

The absence of PTH leads to decreased remodeling in both trabecular and cortical bone and a consequent increase in BMD. Peripheral quantitative computed tomography shows increased cortical and trabecular volume and decreased cortical porosity, without evidence of changes in bone strength **(A)** (150). Histomorphometry shows increased trabecular volume and width, with the maintenance of the trabecular number and spacing, decreased bone formation, and a lower bone resorption rate, which indicates a deep reduction in bone turnover rate in hypoparathyroidism **(A)** (151,152). Retrospective epidemiological studies have shown conflicting results concerning fractures of the superior limbs, showing a lower risk of fracture of the proximal humerus in postsurgical hypoparathyroidism and increased risk of fracture of the forearm in nonsurgical hypoparathyroidism **(A)** (34,145). The risk of fractures, in general, has been described as comparable to that in control populations (34,145). A pioneer prospective study with radiological assessment of vertebral fractures has shown increased morphometric fractures in women with postsurgical hypoparathyroidism **(B)** (153), a finding confirmed by other authors (154). Monitoring of bone mass using dual-energy X-ray absorptiometry (DXA) has little value during long-term follow-up of patients with hypoparathyroidism, noting that the BMD tends to increase with the duration of the disease (155). With the recent findings of a higher prevalence of morphometric vertebral fractures in patients with hypoparathyroidism, periodic radiologic monitoring of the spine is recommended in patients at risk.

**Highlights SBEM:** Chronic complications of hypoparathyroidism are associated with the progression of the disease and its treatment, and include renal manifestations (hypercalciuria, nephrocalcinosis, nephrolithiasis, and renal insufficiency), cataract, cerebral calcifications, cognitive and affective manifestations, changes in quality of life, vertebral fractures, and increased cardiovascular risk **(A)**. Periodic monitoring of such complications is recommended and should be individualized **(D)**.

## CONCLUSIONS

Hypoparathyroidism is a neglected disease, and the scarcity of data regarding this condition restricts potential recommendations for its ideal management. The most common etiology of hypoparathyroidism is the surgical resection of the parathyroids and, therefore, the risk of postsurgical hypoparathyroidism should always be considered when thyroid and parathyroid surgeries are recommended, and the extent of these procedures is planned. From a surgical point of view, the maintenance of intact parathyroids in place is the most important factor in preventing hypoparathyroidism.

Acute hypocalcemia is associated with frequent symptoms including paresthesias, cramps, pain, and muscle weakness, seizures, tetany, and laryngospasm, while chronic hypocalcemia may present with fewer manifestations. Hypoparathyroidism is suspected on clinical grounds but is diagnosed based on laboratory tests indicating inappropriately low PTH levels in the presence of hypocalcemia.

The treatment of hypoparathyroidism is focused on correcting hypocalcemia and hyperphosphatemia, reducing symptoms, and preventing chronic complications. The treatment goal is to maintain the patient asymptomatic with a total calcium level close to the lower limit of the normal range, with the administration of active vitamin D and calcium salts. Special situations such as pregnancy and lactation require frequent and individualized monitoring.

The chronic complications of hypoparathyroidism are related to the disease progression and its treatment and include renal, ocular, cardiovascular, bone, and neuropsychiatric manifestations. Monitoring these complications is recommended. More studies are needed to evaluate their progression and response to treatment in order to improve the management of hypoparathyroidism.
